# Developing of the Computer Method for Annotation of Bacterial Genes

**DOI:** 10.1155/2015/635437

**Published:** 2015-12-06

**Authors:** Mikhail A. Golyshev, Eugene V. Korotkov

**Affiliations:** ^1^Bioinformatics Laboratory, Centre of Bioengineering, Russian Academy of Sciences, Prospekt 60-letiya Oktyabrya 7/1, Moscow 117312, Russia; ^2^Cybernetics Department, National Research Nuclear University “MEPhI”, Kashirskoe shosse 31, Moscow 115409, Russia

## Abstract

Over the last years a great number of bacterial genomes were sequenced. Now one of the most important challenges of computational genomics is the functional annotation of nucleic acid sequences. In this study we presented the computational method and the annotation system for predicting biological functions using phylogenetic profiles. The phylogenetic profile of a gene was created by way of searching for similarities between the nucleotide sequence of the gene and 1204 reference genomes, with further estimation of the statistical significance of found similarities. The profiles of the genes with known functions were used for prediction of possible functions and functional groups for the new genes. We conducted the functional annotation for genes from 104 bacterial genomes and compared the functions predicted by our system with the already known functions. For the genes that have already been annotated, the known function matched the function we predicted in 63% of the time, and in 86% of the time the known function was found within the top five predicted functions. Besides, our system increased the share of annotated genes by 19%. The developed system may be used as an alternative or complementary system to the current annotation systems.

## 1. Introduction

Recent advances in genome sequencing have provided access to a wide variety of nucleic acid sequences [1]. Thousands of complete bacterial genomes, as well as numerous eukaryotic genomes, are now available for use. But to effectively apply this knowledge, we must understand the functions of genes in cells, which makes functional characterization, that is, annotation of the already sequenced genes, our top priority [2]. There are two methods to solve this task. The first one is* in vitro*, the experimental biological approach, which allows us to receive the most reliable information about the functions of genes and other sequences [3]. However, these researches are quite time-consuming and expensive.* In silico* approach is the other option: computer-based annotation is rather low-cost and the results can be obtained much faster. Yet the reliability is not high compared to the experimental approach. Besides, there are genes which cannot be annotated with the computer approach, and their share in bacterial genomes, though varying for different genomes, averages 45% [4]. Our purpose is to develop new mathematical and computational techniques in order to increase the share of annotated genomes and improve the annotation reliability [5, 6].

The bacterial genes computer annotation is based on one main principle: if two sequences are similar, the probability of their biological functions being similar is very high. This idea underlies all of the currently used mathematical annotation methods [7, 8] of which the most widespread are those based on the heuristic similarity search algorithm, multiple sequence alignment, hidden Markov model (HMM), and complex systems combining several methods. These methods were used to assign functions to nearly 60% of sequenced bacterial genes, while around 40% are not yet characterized. Let us examine the main computer annotation methods in more detail.

*(1) Dynamic Programming and Heuristic Algorithms*. The main principle behind the annotation is as follows: if a known sequence in a database is similar to the one under study, their functions are likely to be similar too. Methods used to detect similarities between nucleotide sequences include global and local alignment, both of which are based on dynamic programming [9, 10]. These methods are the most precise ones but are not very efficient due to their extensive computational complexity. Therefore, the heuristic programming tools for pairwise alignment such as BLAST [11] and FASTA [12] with various Expect value thresholds are more widespread. As a source of sequences with known functions they use the following databases: RefSeq [13], GenBank [14], KEGG Genes [15], UniProt [16], and Swiss-Prot [17]. Compared to the dynamic programming, however, the heuristic algorithms discover much fewer significant alignments. At the same time, this is the only approach allowing us to analyze all the gene sequences available so far. 

*(2) HMM-Based Systems*. Pfam [18] and TIGRfam [19] are both protein families databases containing multiple alignments, HMM models, and related information for automatic classification and annotation of new proteins. The search for the most probable models is carried out with HMMER3 [18] or PSI-BLAST [20] software tools. To annotate genes using HMM, it is necessary to form the training and validation gene sets, train the HMM models, and conduct cross-validation. Then the best match between the HMM and the gene under study is used for functional annotation. This approach inherits all of the features, advantages, and disadvantages of machine learning: importance of forming original samples correctly, avoiding system retraining, and so forth. At the same time, the quality of functions prediction with HMM is much higher than in some machine learning algorithms [21].

*(3) Phylogeny-Based Methods*. One of these methods uses the COG database [22], which contains clusters of orthologous genes. Three or more genes are grouped into one cluster if they are found in different genomes and are more homologous to each other than to other genes in these genomes. Currently there are about five thousand COG clusters with known biological functions. The main idea is that orthologous genes are likely to have the same biological functions. The method used to define such functions is similar to the methods described above. To annotate a gene, initially there is a database created containing clusters of orthologs of known genes. Further, the functions of the gene under study as well as its COG cluster are defined by way of searching for similarities between this gene and the known genes from the database. The sequences are compared by searching for significant alignments with the BLAST software. One of the disadvantages of the approach is the need to analyze a significant number of organisms before a phylogenetic tree and COG clusters can be created; the other one is that to conduct the search for significant alignments the heuristic tools are used, and they cannot guarantee that all statistically significant alignments are discovered.

*(4) Pipelines*. InterPro [23] is a system that uses the protein families database with known functions, signatures, and Gene Ontology [24] (GO) terms to determine features of new proteins. InterPro contains 11 different databases: Pfam, TIGRfam, SUPERFAMILY, and others. For search and annotation the InterProScan tool is used [25].

IMG(-ER) [26] is a system for automatic annotation of new genomes and expert functions review. It includes native IMG terms derived from Pfam, TIGRfam, COG, Swiss-Prot, GO, and KEGG and is used for annotation of completely new genomes and for complementation of existing annotations. The database contains more than four thousand various gene functions; about 20% of all genes are covered by IMG terms.

JCVI [27] is a system for structural and functional annotation of genes. Functional annotation is based on BLAST, RPS-BLAST, HMM, and other systems for homology search between nucleotide sequences. As a result of annotation, the gene is assigned a name, symbol, GO terms, EC number, and JCVI functional role categories.

RAST [28] is a fully automated service for annotating bacterial and archaeal genomes. It uses manually curated subsystems of functional roles and protein families (FIGfams) largely derived from the subsystems. This service is developed by the SEED project, which also provides convenient tools for viewing and analyzing results of the annotations.

GenDB [29] is an open source project that provides a web interface and API for gene annotation. For functional annotation it uses BLAST, HMMER, InterProScan, and other prediction tools.

Although by using several annotation methods we can increase the number of genes with predicted functions, complex systems inherit features and drawbacks of their subsystems. Besides, it is sometimes difficult to choose between the results from different algorithms.

*(5) Phylogenetic Profiles*. When the similarity between two nucleic or amino acid sequences is not strong (usually that means below 70%), we cannot be sure that these sequences have the same biological roles notwithstanding the number of similarities found. However, we shall consider the fact that not a separate gene but a combination of genes involved in a genetic process is relevant for the viability of bacteria. This means that genes found in one and the same combination in different bacterial genomes are most probably involved in the same genetic process. Hence, the information about the gene under study being involved in a group of genes present in genomes of different bacteria may be critically important for prediction of its function.

To obtain this information, we form the so-called phylogenetic profiles [30]. They are created for every gene of the bacterial genome using the following method. First, certain genome sequences are selected, which we will call the reference group. Then a phylogenetic profile is built for every gene in these sequences; this profile is a vector of ones and zeros with the length equaling the size of the reference group. Thus, every gene from the group matches 0 or 1 in the corresponding phylogenetic profile: a zero means the bacterial genome contains no homolog for the gene under study; if a similar gene is found, the entry is a one.

After constructing profiles for the reference group, we build one for the gene under study. Using a similarity metric we can now compare the profiles. If the gene under study is part of a combination involved in one genetic process, its profile will be similar to one or several profiles in the reference group. Otherwise no similarities will be found.

This approach was first used by Pellegrini for protein sequences [31] and was sufficiently developed over the last ten years in terms of the creation, comparison, and analysis of phylogenetic profiles. Particularly, the concept of using real vectors or matrices instead of binary vectors was developed. Also, various approaches to comparison of phylogenetic profiles were suggested, such as the mutual information approach, Jaccard coefficient, Pearson correlation, and hypergeometric distribution. The detailed review of the approaches was given in studies [32, 33].

However, the results very much depend on the similarity search method used. In this study, we used the phylogeny-based method, though it is a little amended. Firstly, with the help of BLAST we searched for homologs with different values of reward and penalty, which ensured the reasonable search speed and allowed us to find a large number of local alignments. Secondly, we used the dynamic programming algorithm [9] with the PuPy substitution matrix [34] to see if a statistically significant global alignment could be found where BLAST had discovered a local one. The reason we looked for global alignments only was that local alignments often indicate partial similarities, not the whole gene homologs. To define statistical significance of a global alignment, the Monte Carlo method was used [35]. Thirdly, we compared the annotated gene to the bacterial genomes, not to single genes, which saved us from mistakes associated with the structural annotation of bacteria, that is, with genes demarcation. Following this analysis, a phylogenetic profile was built for every gene under study, which was then compared to the profiles of the reference group genes. Our study resulted in annotation of an additional 19% of genes which could not be annotated with any of the previously used methods. At the same time, we were unable to assign statistically valid functions to 9% of the genes.

## 2. Materials and Methods

### 2.1. Phylogenetic Profiles

Phylogenetic profiles are used to create sets of genes that are involved in the same genetic process. This approach was first applied in 1998 by Pellegrini and his colleagues [31]. To create a phylogenetic profile of a gene, it is necessary to form a binary vector as follows: if a gene has been detected in the *i*th genome, the *i*th position of the vector contains 1; if there is no gene found, it is 0. We assume that the genes involved in the same genetic process will have similar phylogenetic profiles constructed from the same set of reference genomes. The assumption is derived from the fact that the gene normally performs its function not alone but in conjunction with other genes as part of one metabolic pathway. In the course of evolution this process is inherited by different organisms; as a result, more functional groups emerge containing genes of similar profiles [36].

In this paper, for the predicted function we take one of the most probable predicted functions from the gene's functional group. As you can see, the phylogenetic approach does not use direct comparison of the coding sequences of genes against each other but takes into account the cooccurrence of certain genes in the genomes. So, this approach can supplement the annotation methods discussed in the previous section and predict functions for those genes, for which the best similarity is significantly lower than 70%.

### 2.2. The Method Description

**Figure 1:**
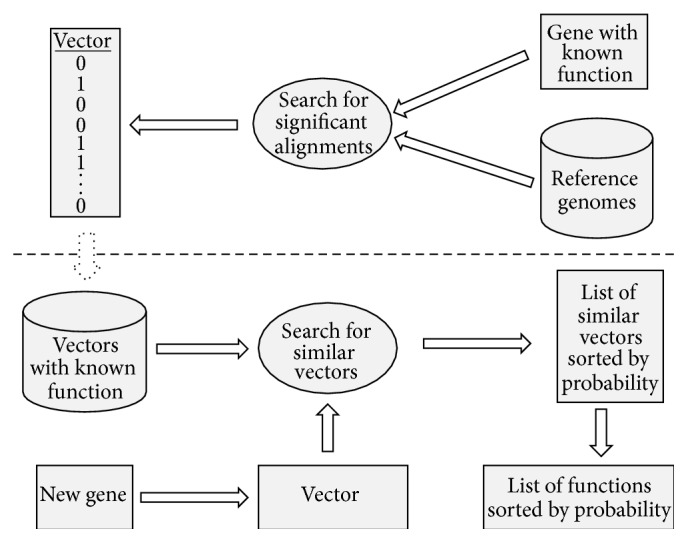
Creation of the phylogenetic profiles database for genes with known functions. Function prediction for a new gene.

Our work in this study had two stages: creation of a database containing phylogenetic profiles of genes with known functions and prediction of the functions for genes using the previously created database (Figure 1).

To create a phylogenetic profile of a gene, it is necessary to determine a set of reference genomes. As of this writing, there were more than 2,100 bacterial genomes sequenced; however, using close genomes, for example, strains of one organism, impairs precision of predictions because occurrences of the gene in such genomes are not independent. So from all bacterial genomes we only selected 1,204 as reference genomes. To create the database of phylogenetic profiles, we used all the genes with known functions from the 1,204 reference genomes: 3.7 million genes in total.

The major task in the database creation process was to determine the similarity significance for each pair of genes and genomes. First, we used BLAST with different options to search for significant local alignments. After that we extended the found local alignments to global alignments and for each global alignment we calculated scores *F* of dynamic programming (Needleman-Wunsch algorithm [9]) using the PuPy matrix. Using the Monte Carlo method and (1), we calculated statistical significance for each global alignment on the assumption that the distribution of the score *F* was normal [37]:

(1) $$\begin{array}{c} Z=\frac{F-M\left(F\right)}{\sqrt{D\left(F\right)}}, \end{array}$$

where *F* is the score of alignment and *M*(*F*) and *D*(*F*) are the sample mean and sample variance of the random value *F*. The sequences sample was created from the original sequence by randomly shuffling its symbols. *M*(*F*) and *D*(*F*) were calculated on the samples with size 1000.

Further, we created binary vectors for each gene by the following rule: we assigned 1 to the *i*th element of the vector if the statistical significance of the global alignment between the gene and the *i*th genome exceeded the chosen minimal value and 0 if no similarity was found or if its significance did not exceed the chosen minimal value. Therefore, for each gene we created a binary vector with length *N*, where *N* is the number of referent genomes. We chose the minimal value of statistical significance *Z* = 5.0, so that the probability to find more than one 1 for random sequences was 5%.

Since the names of the same functions may vary in different annotation systems, we unified them by using the Gene Ontology (GO) terms. As a result, the predicted functions in our system are represented as GO terms.

To predict a function, we first create a binary vector for the gene in the same manner as when creating the database of known functions, after which we search for similar vectors in this database using the probability measure that will be described below. Let *N* be the size of the reference group and let the vector length, *n*_1_, be the number of 1 in the vector (i.e., in the phylogenetic profile) of the first gene, *n*_2_ the number of 1 in the vector of the second gene, and *n*_12_ the number of common 1 (i.e., placed in the same positions) in the first and second genes. As a measure of similarity between two vectors, we chose the probability *P* of observing *n*_12_ or greater cooccurrences between two profiles purely by chance. As is known, the random variable of common 1 follows the hypergeometric distribution [38]; hence the probability *P* can be calculated by

(2) $$\begin{array}{c} P\left(\;n\geq n_{12}\;\right)=\overset{\min\left(n_{1},n_{2}\right)}{\underset{k=n_{12}}{\sum }}\frac{C_{n_{1}}^{k}\times C_{N-n_{1}}^{n_{2}-k}}{C_{N}^{n_{2}}}, \end{array}$$

where *C*_*n*_^*k*^ = *n*!/*k*!(*n* − *k*)! is the number of *k*-combinations from the given set of *n* elements.

Vectors of the genes, the probability *P* for which did not exceed the chosen threshold, participate in determining the potential function of the annotated gene. The result of the prediction is a list of possible functions, sorted by the probability *P*. For phylogenetic profiles filtering, we chose the *P*_0_ threshold of 10^−7^. The vector pairs with *P* > *P*_0_ are considered different. We tested the selected threshold on a set of random vectors: the selected *P*_0_ value provides such level of significance, in which of 10^7^ comparisons of two random phylogenetic profiles no more than one has the level of *P* < *P*_0_.

## 3. Results

### 3.1. Comparison of the Current Work to Previously Conducted Annotations

To evaluate the quality of the developed method, we used it to predict possible functions for the genomes which had already been annotated. Since the system database already contained genes from these genomes, for testing purposes we excluded them from the reference group. The method detects a functionally linked group of genes rather than the one most probable function. That is why we compare the known function not to the single predicted one but to the first *K* of more probable functions. Below we describe the approach in more detail.

Of 1204 reference genomes we selected at random 104 bacterial genomes from various families. For every genome, we defined the method it was formerly annotated with and then grouped the genomes accordingly (Table 1). It was essential so that we could afterwards compare our results to the results obtained from the previous annotations based on different methods.

The system presents the predicted function as a set of Gene Ontology terms. Let us see what the GO terms are in more detail. Each term may belong to one of the three domains: cellular component (C), molecular function (F), and biological process (P). Hence, every function may be presented as a set of terms from these domains, though not necessarily from all three of them at once. It is worth noting that GO terms in each domain are structured as a tree, where each term is a leaf or an internal vertex. We were mostly interested in molecular functions of genes; therefore in this study we will only cover results for terms of this type (F); however, similar results were obtained for every type (C, F, and P) separately and for the combination of all three together. To compare sets of terms, we used two approaches:* perfect match*, when all the terms should match for the sets to be equal, and* fuzzy match*, when the sets are considered equal if at least one pair of terms match one another.

**Figure 2:**
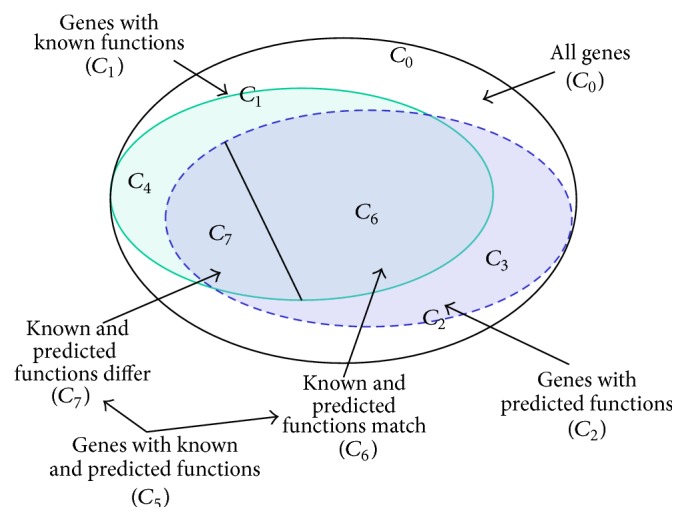
Subsets of genes under study.

By the known function we will mean the previously annotated function, and by the predicted function, the one obtained in this study. To define the system characteristics, we introduced subsets, which are displayed in Figure 2 and described in detail in Table 2. Since the annotation results are presented as a list of possible functions, we consider the functions equal if the known function is found within the first *N* most probable predicted functions. The list was arranged by probability* P* (see (2) below); for this study we take *N* = 5.

To evaluate the precision of predictions, we split the* C*_5_ set into two subsets. Let the* C*_6_ set be a subset of genes for which the known function was found within the top five (*N* = 5) predicted functions. Therefore, *C*_7_ = *C*_5_ − *C*_6_ is a subset of those genes from* C*_5_ for which the known function differs from the predicted function (the known function was not found within the top five predicted functions).

In Table 2, we would like to highlight the two sets and two subsets of genes which are essential for estimating the quality characteristics of our annotation system in comparison with the annotations that have been made previously. These are sets* C*_3_ and* C*_4_ and subsets* C*_6_ and* C*_7_. The* C*_3_ set contains the genes that have predicted functions, but no known functions. The* C*_4_ set contains the genes that have known functions, but no predicted functions. The* C*_6_ and* C*_7_ subsets were defined in the previous paragraph.

**Figure 3:**
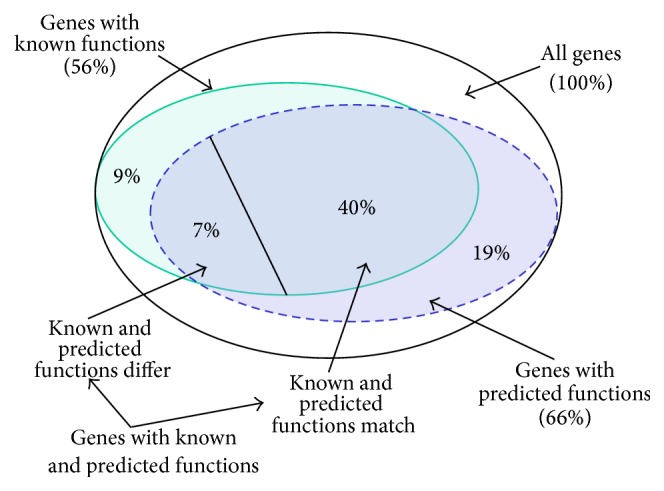
Visualization of annotation results.

This section contains prediction results grouped by method of their original annotation and by method of comparison of their known function with the predicted ones. In all tables we define the size of the *C*_*i*_ sets as *N*_*i*_. Tables 3 and 4 show the share of various gene sets in the total number of genes: these are the set of previously annotated genes and the set of genes annotated with our system, as well as their intersections and subsets. The obtained results can be visualized with the diagram in Figure 3 (the* perfect match* method of functions comparison is used).

It is clear that the share of genes from the* C*_3_ set varies from 16.9% to 21.4% and averages 19% (Table 3). The share of genes from the* C*_4_ set varies from 6.8% to 11.3% and averages 9%. To determine the equality of known and predicted functions, we used the two above-described ways,* perfect match* and* fuzzy match* (Table 4). The share of genes from the* C*_6_ set varies from 37.7% to 44.4% and averages 40% (Table 4). The share of genes from the* C*_7_ set varies from 3.8% to 8.5% and averages 7%. As you can see from Table 4, these results vary slightly depending on the comparison approach (*perfect match* or* fuzzy match*). The major difference between the known and predicted functions (i.e., the maximum ratio of* N*_7_/*N*_6_) is observed for the group of genes defined in Table 1 as GRP_4.

It is also interesting to estimate the precision of predictions for the top one (*N* = 1) function of the genes from the* C*_3_ set. For this purpose we analyzed the genes from the* C*_5_ set (which consists of the genes that have both known and predicted functions) and found for each gene the minimum size of the predicted functions list so that it contained the known function. This dependence in terms of percentage points is presented in Table 5. The size of the* C*_5_ set is designated as 100%; each row shows the share of each place in the list where the known function was found. As can be seen from Table 5, the known function was found on the top of the predicted functions list in 63% of the time and in Positions 2 to 5 in 23% of the time; 13% of cases accrued to Position 6 and higher. These results show that when we use the most probable predicted function, the precision to predict the known function is 63%. Therefore, we can conclude that precision for genes from the* C*_3_ set may be the same.

These results also justify the choice of *N* = 5 for comparing the biological functions for the* C*_5_ set genes (*C*_6_ + *C*_7_). As you can see from Table 5, the share of exactly predicted functions stops increasing notably at *N* = 3 and reaches saturation at *N* = 5.

### 3.2. Results of Metabolic Pathways Prediction

As is known, each gene may be part of one or several metabolic pathways. In other words, the gene may be functionally linked with one or several groups of genes. Based on this statement, we created functional groups for each gene. This statement may be verified by calculating the share of common functions between a metabolic pathway and a predicted functional group. This measure may be an additional quality characteristic for the system. For each gene, we determined the number of predicted functions that were involved in its metabolic pathway. The information about metabolic pathways was received from the KEGG database and processed in the following way:

1. Since metabolic processes can be quite extensive, we filtered out those containing more than 30 genes.
2. We consider only those genes that were involved in at least one metabolic process described above.
3. If a gene was involved in more than one metabolic pathway, we selected for it the pathway with the smallest number of genes.

For each gene we formed two lists of most probable functions with lengths *Q* and 2*Q*, where *Q* is the length of the metabolic pathway of the gene. After that, we calculated the share of metabolic pathway functions contained in each of these lists. Accordingly, the higher the share is, the better the predicted functions group defines the metabolic pathway.

About 90,000 genes from 375,000 genes under study were involved in metabolic pathways from the KEGG database. We chose 70,000 genes that were involved in the filtered metabolic pathways described above. The metabolic pathways were grouped by the number of contained functions; the results for each group were averaged by the size of the group and can be found in Table 6. As you can see, for the* fuzzy match* approach the coverage of the *Q* length metabolic pathway by the list of top *Q* predicted functions is about 0.5, and for the list of top 2*Q* predicted functions it is about 0.6. At the same time, for the* perfect match* approach we evidence high dependency on the length of a metabolic pathway, and the coverage strongly decreases with the increase of the length. If the length of a metabolic pathway is in the range from 1 to 4, the coverage is about 0.4; for pathways with the length between 25 and 30 the coverage is about 0.1.

The results of annotations for genes under study can be freely accessed at http://genefunction.ru/public_results/.

## 4. Discussion

First of all, it is interesting to consider the genes for which functions predicted in our study differ from the known functions. They fall into the subset of genes which we defined as* C*_7_ in Table 2 and Figure 2. The share of this set is 7% from the total number of the genes under study (Figure 3). The difference can be explained by the fixed size of the top predicted functions for each gene. To compare them with the known functions we use the top five predicted functions sorted by probability *P*. As you can see from Table 5, a known function was found within the top five predicted functions for 86% of the genes. For 14% the five best predicted candidates did not contain an already known function. This may occur in three cases. Firstly, the genes may be involved in several metabolic pathways with different functions (i.e., functions of the gene in these pathways are different). If one of these metabolic pathways is more widespread in genomes under study than the others, the function of the gene in this pathway may be predicted as more probable; thus the previously predicted (known) function may not be found among the top five predicted functions. Secondly, the gene may have a mutated copy (paralog), which takes part in a different genetic process. Such paralog may participate in a metabolic process that can be found in a greater number of reference bacterial genomes than the metabolic process in which the original gene we study participates. Thirdly, there might be a mistake made in previous annotations, but the probability of that to happen is very small, which may be explained by the high level of similarity between sequences in the previous annotations.

It is also interesting to consider the* C*_4_ set which contains genes for which no predicted functions were found in the present work. The share of such genes is 9% of the total number of analyzed genes. There are two reasons to explain the absence of predicted functions for these genes. The first is that the search for similarities in this work was performed by comparing the nucleotide sequences rather than the amino acid sequences. Some significant similarities of the amino acid sequences may appear insignificant on the nucleotide level, and their statistical value will be below the threshold level. Secondly, this may be explained by the specific feature of the approach: to create a group of related genes it is necessary to find similar vectors with a sufficient number of 1; that is, the gene must be found in a sufficient number of different genomes. In most cases when a group cannot be created, it is because of few 1 in the profile of the gene rather than due to the absence of similar vectors.

The most successful result of our work is the* C*_3_ subset of genes for which there were no previously predicted functions before our study; the share of this set is 19% of all genes that have been examined in the present work. The fact that these functions have never been predicted before can be explained by the difference of approaches. The vast majority of the existing annotation methods identifying orthologs use amino acid sequences with the sufficiently high level of similarity only, which allows predicting the equality of their biological functions with great probability: the higher the similarity, the stronger the indication that these sequences are exact orthologs. When the similarity level is lower (though still statistically significant), more potential homologs can be found: the greater part of them are paralogs (mutated copies with unrelated functions), but it is entirely possible that orthologs may also be found among these similarities. To separate one from another, some additional information must be used. In this work, such information is the similarity of phylogenetic profiles. The similarity between the profiles will be significant for orthologs and either missing or statistically less significant for paralogs. Therefore, this additional filtering by phylogenetic profiles allows us to sort out paralogs and to predict biological functions for genes using the similarities not accounted for by the existing annotation methods. We also increased the number of significant similarities by using several cycles of local alignments search with different parameters, including the purine-pyrimidine weight matrix for global alignment. Besides, we compared each gene with whole bacterial genomes rather than with sets of previously selected genes from these genomes. It allowed us to avoid errors during structural annotations, that is, when identifying the gene sequences in the bacterial genomes. To sum it all up, our success in annotating new genes is based on the phylogenetic profiles comparison method, which allowed us to find additional orthologs among a great number of paralogs.

Let us also estimate the precision of biological function predictions for genes from the* C*_3_ set. For this estimation, we use as the prediction result the first function in the list sorted by probability *P*. As you can see in Table 5, the predicted biological functions of 63% of all genes examined in the present work coincide with known functions. It can be expected that the precision of predictions for the* C*_3_ genes will be the same (about 63%). The obtained results look reasonably better in comparison to similar studies; for instance, in a previous study for the* E*.* coli* genome the known function was found on the top of the predicted functions list in 43% of the time and within the top ten in 60% of the time and for the* S*.* cerevisiae* genome the known function was found within the top fifty predicted functions in 60% of the time [39]. However, in our study the known function was on average found within the top five predicted functions in 86% of the time.

Since the developed system first of all determines groups of functionally linked genes and only after that the single functions, we tried to estimate the quality of such functional groups by comparing their functions with those of the genes in metabolic pathways. Comprehensive results can be found in Table 6. A predicted group of size *N* contains about half of the functions of the size* N* metabolic pathway when compared with the* fuzzy match* method. Thus, we can conclude that the coverage of the metabolic pathway increases if we expand the list of the top predicted functions. On this conclusion we agree completely with the previously published studies [40, 41]. We consider these results to be reasonably good. The existing systems of metabolic pathways are based on the readings from publications and literature, while our system uses the nucleotide sequence itself (thus making it possible to create functional groups even if there is no published information for the metabolic pathway of the gene). So it may serve as a very fast preliminary method to create groups of genes taking part in the same genetic process.

We compared directly the annotation, which we did with the help of our system, with the annotation, which can be done by RAST and InterProScan [25, 28]. For this, we chose 10 random bacterial genomes which together contained 32536 genes. All these genes were annotated by RAST, InterProScan, and our system and the annotation results are shown in Table 7 in terms of Table 2. From these results it is seen that our system annotates 9 and 13% of genes additionally to RAST and InterProScan, respectively. At the same time, for 17 and 12% of genes annotated by RAST and InterProScan our system cannot make the annotation. In general the direct analysis gave the results comparable with the results received in the original publications (Figures 2 and 3). We made the estimate of the number of false positives which may be present in the annotation results. To do this, we mixed up the sequence of each of 32536 genes with preservation of the triplet periodicity [42] and then random sequences were annotated as real genes. We received 18% and 1.3% of random sequences annotated using RAST and InterProScan, respectively. In the case of our system, the number of annotated random sequences was about 0.5%. Thus, the annotation done by RAST contains a significant number of false positives and the accuracy of the RAST annotation is very low. The number of false positives for InterProScan and for our system is comparable.

Although the developed system does not make exact predictions of gene functions (the precision is about 63%; see Table 5), it may be used as an alternative or complementation to the existing annotation systems: the existing systems predict functions for genes from sets* C*_4_ and* C*_5_, and our system covers functions for genes from sets* C*_3_ and* C*_5_. Therefore, the use of our system can increase the share of annotated bacterial genes by 19% (by the size of the* C*_3_ set).

## Figures and Tables

**Table 1:** Bacterial genomes grouped by annotation method.

Annotation methods	Number of genomes	Group ID
NCBI, UniProt, TIGRfam, Pfam, PRIAM, KEGG, COG, InterPro, IMG-ER	38	GRP_1

BLAST, homology	28	GRP_2

GenDB, BLAST, COG, COGnitor	7	GRP_3

InterPro(Scan)	5	GRP_4

Total	104	GRP_ALL

**Table 2:** Subsets of genes used to compare the current and previous annotations.

Name	Description
*C*_0_	All genes under study

*C*_1_	The subset of genes from the *C*_0_ set that have known functions

*C*_2_	The subset of genes from the *C*_0_ set that have predicted functions

*C*_3_	The subset of genes from the *C*_2_ set that have predicted functions, but no known functions. *C*_3_ = *C*_2_ − *C*_1_

*C*_4_	The subset of genes from the *C*_1_ set that have known functions, but no predicted functions. *C*_4_ = *C*_1_ − *C*_2_

*C*_5_	The subset of genes from the *C*_0_ set that have both known and predicted functions. *C*_5_ = *C*_1_∩*C*_2_

*C*_6_	The subset of genes from the *C*_5_ set for which the known function was found within the top five predicted functions

*C*_7_	The subset of genes from the *C*_5_ set for which the known function was not found within the top five predicted functions. *C*_7_ = *C*_5_ − *C*_6_

**Table 3:** Shares of *C*_1_–*C*_5_ subsets in the total number of genes (*N*_0_). *N*_1_/*N*_0_ is the share of genes from the *C*_1_ set; *N*_2_/*N*_0_ is the share of genes from the *C*_2_ set; *N*_3_/*N*_0_ is the share of genes from the *C*_3_ set; *N*_4_/*N*_0_ is the share of genes from the *C*_4_ set; *N*_5_/*N*_0_ is the share of genes from the *C*_5_ set.

Group ID	*N*_0_ (number of genes)	*N*_1_/*N*_0_	*N*_2_/*N*_0_	*N*_3_/*N*_0_	*N*_4_/*N*_0_	*N*_5_/*N*_0_
		*C*_1_	*C*_2_	*C*_3_	*C*_4_	*C*_5_
GRP_1	144157	0.551	0.613	0.169	0.113	0.444
GRP_2	82170	0.573	0.668	0.186	0.091	0.482
GRP_3	24657	0.568	0.714	0.214	0.068	0.500
GRP_4	20592	0.549	0.663	0.194	0.080	0.469
GRP_ALL	375151	0.563	0.658	0.186	0.091	0.472

**Table 4:** Comparison of original and predicted functions. *N*_5_/*N*_0_ is the share of genes from the *C*_5_ set (these genes have both known and predicted functions), *N*_6_/*N*_0_ is the share of genes from the *C*_7_ set (genes from *C*_5_ for which the known function and the predicted function are equal), and *N*_7_/*N*_0_ is the share of genes from the *C*_7_ set (genes from *C*_5_ for which the known function differs from the predicted function).

Group ID	*N*_5_/*N*_0_	Perfect match		Fuzzy match	
		*N*_6_/*N*_0_ = *C*_6_	*N*_7_/*N*_0_ = *C*_7_	*N*_6_/*N*_0_ = *C*_6_	*N*_7_/*N*_0_ = *C*_7_
GRP_1	0.444	0.377	0.067	0.401	0.043
GRP_2	0.482	0.420	0.062	0.444	0.038
GRP_3	0.500	0.432	0.068	0.460	0.040
GRP_4	0.469	0.384	0.085	0.419	0.050
GRP_ALL	0.472	0.407	0.065	0.432	0.040

**Table 5:** Distribution of places in the list of predicted functions where known function was found.

Position of the known function in the list	Cumulative percentage of genes	Percentage of genes	Number of genes
1	63.23	63.23	108806
2	77.12	13.89	23894
3	82.06	4.94	8498
4	84.64	2.58	4446
5	86.23	1.59	2743
6	87.36	1.13	1949
7	88.19	0.83	1433
8	88.84	0.65	1127
9	89.40	0.56	962
10	89.87	0.47	783

**Table 6:** The average share of functions from the *Q* length metabolic pathway in the lists of top *Q* and top 2*Q* predicted functions.

Approach	Size of predicted functions list	Metabolic pathways length range (*Q*)					
		1–4	5–9	10–14	15–19	20–24	25–30
Perfect match	*Q*	0.381	0.198	0.162	0.115	0.097	0.090
	2*Q*	0.415	0.235	0.196	0.144	0.125	0.115

Fuzzy match	*Q*	0.549	0.462	0.495	0.440	0.427	0.453
	2*Q*	0.588	0.530	0.572	0.538	0.543	0.578

**Table 7:** Using of RAST and InterProScan for annotation of genes from 10 bacterial genomes. Subsets of genes *C*_3_, *C*_4_, and *C*_5_ are shown (Table 2).

	*C*_4_, %	*C*_5_, %	*C*_3_, %
RAST	17	48	9
InterProScan	12	44	13
